# Experiencing aging or demystifying myths? – impact of different “geriatrics and gerontology” teaching strategies in first year medical students

**DOI:** 10.1186/s12909-017-0872-9

**Published:** 2017-02-08

**Authors:** Alessandra Lamas Granero Lucchetti, Giancarlo Lucchetti, Isabella Noceli de Oliveira, Alexander Moreira-Almeida, Oscarina da Silva Ezequiel

**Affiliations:** 1 Division of Geriatrics, School of Medicine, Federal University of Juiz de Fora, Av. Eugenio do Nascimento s/n, Bairro Dom Bosco, Juiz de Fora, Brazil; 2 School of Medicine, Federal University of Juiz de Fora, Juiz de Fora, Brazil; 3 Department of Medical Education, School of Medicine, Federal University of Juiz de Fora, Juiz de Fora, Brazil

**Keywords:** Medical education, Geriatrics, Medicine, Older people care

## Abstract

**Background:**

With the aging of the population comes a greater need for geriatric and gerontology teaching. However, there is currently a dearth of investigations on the impact of different educational methodologies for teaching in this area early in medical courses. The present study aims to determine the impact of two educational strategies on the topic “Geriatrics and Gerontology” (“experiencing aging” and “myths of aging”) as compared to a control group (no intervention) on the attitudes, empathy and knowledge of first year medical students.

**Methods:**

An intervention-based study in education was conducted at the beginning of the first year of a medical course. Students submitted to educational strategies were compared against students with no intervention. The two strategies were: “Experiencing Aging” – also known as the “aging game” (simulation of the disabilities and physiological changes of aging), and “Myths of Aging” - a knowledge discussion based on a “quiz show”, questioning common myths about aging. All students were assessed on their attitudes towards older persons (Maxwell-Sullivan, UCLA attitudes), empathy (Maxwell-Sullivan), knowledge on facts and positive view about aging (Palmore), and cognitive knowledge. Data were analysed using Student’s *t*, Chi-squared or ANOVA tests.

**Results:**

A total of 230 students were assessed. The “experiencing aging” intervention was associated with improvement in empathy but worsening of attitude. The “myths of aging” intervention was associated with an improved attitude overall and positive view about aging but with no change in empathy towards older persons.

**Conclusion:**

Educational strategies can influence the attitudes and empathy of students, leading to different outcomes. These data highlight the importance of assessing the outcomes of educational strategies in medical teaching to ascertain in what manner (how), situations (when) and settings (where) these activities should be introduced.

**Electronic supplementary material:**

The online version of this article (doi:10.1186/s12909-017-0872-9) contains supplementary material, which is available to authorized users.

## Background

Population aging is a global reality, with estimates projecting that 22% of the world population will be older adults by 2050 [1]. This rapid demographic transition poses new challenges in medicine, such as managing comorbidities, chronic diseases, understanding physiological changes of aging, recognizing different pharmacokinetics and pharmacodynamics and their implications for prescribing, institutionalization, palliative care, as well as ethical and legal dilemmas [2].

This scenario means that medical students and doctors, irrespective of specialty, need to develop specific Geriatrics and Gerontology competencies to meet the health needs of the older population [3–5]. To this end, many international medical schools have proposed a curriculum which covers these areas [6, 7] along with guidelines requiring a minimum geriatrics content for medical students [2].

However, a consensus is currently lacking on how and when the subject should be introduced into medical training courses. Tullo et al. [8] conducted a systematic review assessing “Geriatrics and Gerontology” teaching strategies for undergraduate medical students. Nevertheless, the results were mixed and further evidence is required on the use of these strategies to modify students’ behavior. Another noteworthy point is that, even after specific educational interventions, students’ attitudes toward elderly worsened over ensuing years, pointing to the need for longitudinal exposures which begin early in the curriculum [9].

The reasons for such worsening are not totally understood. The most probable one is the fact that there is a stereotype against older people (ageism) by physicians and health professionals and, this stereotyped view, is taught to the students during the medical course. In other words, the older person is viewed as cognitive impaired, physical dependent and with emotional problems [10]. A recent systematic review [11] showed that students show little interest in the area of geriatrics driven by the low exposure and complexity of older patients, low financial return and low status, preferring careers which have acute somatic illness than chronic ones. Same results were found by Bagri et al. [12] who found that medical students were depressed by the decline and death of their older patients, were concerned about patients’ unrealistic expectations and opportunities for litigation, felt unsure how to handle ethical dilemmas, and found communicating with older adults to be enjoyable but time consuming and challenging.

In this context, discussions on Geriatrics and Gerontology teaching should consider what, how and when to teach, since teaching and learning regimes (TLRs) can have diverse results. On one hand, some participants could embrace the strategy positively and use it to develop their professional practice. On the other hand, some could express dissatisfaction or unease with it [13]. These challenges may help in the identification on how new strategies could influence the attitude and empathy of undergraduates toward the elderly at an early stage, given that they can influence future changes in behavior of these professionals.

Considering Constructivism as the “theoretical framework” that supports the pedagogical decisions of our medical school curriculum, we felt the necessity of using active strategies in the early phases of the medical course in order to discuss the questions related to aging. For that purpose, ludic strategies, which are a type of experiential learning where learner “engages in some activity, looks back at the activity critically, abstracts some useful insight from the analysis and puts the results to work “were chosen [14, 15]. These strategies are based in a transformative learning theory, which is founded on both humanist and constructivist perspectives [16]. According to Mezirow [17]: *“Transformative learning is learning that transforms problematic frames of reference—sets of fixed assumptions and expectations (habits of mind, meaning perspectives, mindsets)—to make them more inclusive, discriminating, open, reflective, and emotionally able to change”*

There are many types of games used in medical education, such as virtual environments, alternative reality games, simulations and social-cooperative play. A number of studies have been suggesting beneficial effects of using educational games in learning. However, a recent systematic review [18] was inconclusive and asked for additional and better designed studies to assess the effectiveness of these games. Particularly in the field of geriatrics, other systematic review [14] showed that the most commonly used strategies in the scientific literature was the “aging game” (simulation of the disabilities and physiological changes of aging), although results have been conflicting. Other studies have also used interactive games and competitions (social-cooperative play).

Although there is an increasing interest in active learning through educational games and in the necessity of teaching students about geriatrics and in changing their attitudes towards older adults, there are few studies which assessed and compared how these educational strategies could impact first-year medical students. If we could change students’ views in the beginning of the course, it may be possible to reverse the ageism and waken their interest to follow a career in geriatrics.

Therefore, the objective of the present study was to investigate the impact of two educational strategies on the subject “Geriatrics and Gerontology” (a simulated strategy - “experiencing aging” and a social-cooperative play - “myths of aging”), as compared to a control group (not receiving intervention), on the attitudes, empathy and knowledge of first year medical students.

## Methods

### Type of study and setting

An intervention-based quasi-experimental study in education was conducted at the Federal University of Juiz de Fora (UFJF), Juiz de Fora, Brazil, between July 2014 and July 2015.

### Participants

During the first and second semesters of 2014, and the first semester of 2015, all students officially enrolled on the first period of the UFJF medical course were invited to take part in the study. In a pre-defined day, during a first-year course called “Integrative themes in Clinical Practice”, two faculty members (from the Division of Geriatrics) clarified the objectives of the activity, explained about the study and invited students to participate. All students who voluntarily participated in the educational activities proposed and that signed the consent form were included.

### Instruments

The self-report questionnaire (Additional file 1) applied was based on previous studies on education in geriatrics [8], took 25 min to complete, were provided in Portuguese, and contained the following instruments:

- Questionnaires collecting sociodemographic data: gender, age, family income and semester of course.
- Basic knowledge in geriatrics: instrument devised by the researchers for assessing cognitive knowledge (using 10 multiple-choice questions on theoretical content taken from Brazilian public admissions exams for geriatricians). The content of these questions includes: inappropriate prescribing, frailty, delirium, dementia, falls, aging epidemiology, physiological changes with aging and comprehensive geriatric assessment.
- UCLA Geriatric Attitudes Test [19]: test used worldwide for assessing the attitudes of medical students and residents toward elderly patients. This instrument comprises 14 questions using a 5-point Likert response format (1 = totally disagree to 5 = totally agree). The higher the score on the scale, the greater number of positive attitudes held toward elderly people. Some example questions include: “Most old people are pleasant to be with”, “As people grow older, they become less organized and more confused”. In the present study, we found a Cronbach’s alpha of 0.618.
- Facts about aging (Palmore-FAQ-1): a test used worldwide for assessing the knowledge of medical students and residents about elderly patients. The instrument comprises 25 multiple choice questions with four possible answers. An example of a question includes: “Happiness among old people is: (a) rare, (b) less common than among younger people, (c) about as common as among younger people, (d) more common than younger people. The analysis can be done in two ways: tallying only the correct answers - for example, the answer for the question was letter (c) - or interpreting whether students have a more positive or negative view about old people (If the student answered letters (a) or (b) he/she has a more negative view about old people, if the student answered letter (d) a more positive view and if the student answered letter (c) a neutral view [20]. In the present study, we found a Cronbach’s alpha of 0.711.
- Modified Maxwell-Sullivan attitudes toward the elderly scale [21]: this instrument assesses attitudes toward the elderly (8 questions) and empathy (3 questions). Students mark answers on a 5-point Likert response format (1 = strongly agree to 5 = strongly disagree). Some example questions include: (a) attitude: “Treatment of elderly is hopeless”, “Treatment of elderly is too time-consuming”; (b) Empathy: “I can truly empathize with older patients”, “I understand what it feels like to have problems with aging”. In the present study, we found a Cronbach’s alpha ranging from 0.638 (attitudes) to 0.739 (empathy).

### Data collection

**Fig. 1:**
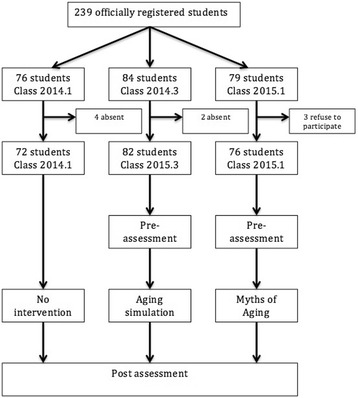
Flowchart: Participant selection process

Two educational strategies were introduced (“experiencing aging” or “myths of aging”) among first year medical students at the beginning of the second semester of the 2014 course – 2014.3 Group (“experiencing aging”) and at the beginning of the first semester of the 2015 course – 2015.1 Group (“myths of aging”). The groups were compared against a Control Group with no intervention (first semester of 2014 – 2014.1 Group) (Fig. 1: Flowchart). The interventions were performed during the first class (first week of medicine course) of a 15-h compulsory course. There was no difference in the classes as cohorts, since they are all first-year students from the same university and with similar admittance methods.

Each intervention was 02 (two) hours’ long and students were split into groups of 25–30. Prior to commencing the intervention, the participants filled out questionnaires (pre-test) and after the intervention completed the questionnaire for a second time (post-test).

For control assessment, the same instruments were applied to students from Group 2014.1. Since the control group has not been submitted to the intervention and the time gap between questionnaires was short (approximately 2 h), they only filled out the questionnaire once and this questionnaire was comparable to a post-test. The comparison among groups employed the same methodology adopted in previous studies [14, 22].

### Intervention

- Experiencing aging (“Aging Game”): students (total 25–30) visited 5 different stations for approximately 20 min each. Through dramatization (“simulation”), the students simulated what it feels like to be an elderly person with several aging-related physiological and pathological impairments. This intervention was based on previous studies [14, 22] and entailed activities such as: Walking difficulties: attachment of weights to legs and walking while negotiating obstacles placed on the floor; Visual problems: glasses simulating visual impairments (e.g. cataracts and glaucoma); Hearing problems: headphones introducing background noise and cotton plugging ears; among others.
- Myths of aging: this activity was devised by the National League of Nursing of the United States and adapted for the Brazilian milieu and medical students. This is a game type activity whose purpose is to help students recognize and deconstruct myths associated with aging and involve them in a discussion about myths and attitudes towards older people. Incorporating a quiz show design (“Jeopardy!^™^” format), it includes topics ranging from physical health to psychosocial issues and sexuality [23]. In the present study, each classroom had a total of 25–30 students divided into three teams [24].

### Statistical Analysis

The descriptive analysis was performed using measures of absolute and relative frequency for categorical variables, as well as mean and standard deviation for continuous variables. The intervention and no-intervention groups were compared at baseline using the Chi-squared (for categorical variables) and ANOVA tests (for continuous variables).

The groups submitted to the educational interventions were assessed pre and post intervention to measure impact, i.e. students served as their own controls for the scales applied (UCLA, Palmore, Knowledge, Empathy, Attitude). A repeated measures paired *t*-test was used for this evaluation. In this case, we applied Bonferroni's correction due to multiple comparisons.

ANOVA for independent measures and the post-hoc Tukey test were used to compare the three groups after the interventions (“experiencing aging”, “myths of aging” and “no intervention”) for attitudes, empathy and knowledge. Since some demographic characteristics (gender and age) differed among groups, we have also conducted an ANCOVA with age and gender as covariates.

Effect sizes (r) were added to all analyses based on the following formulae: (a) t-tests: *r* = √[t^2^/(t^2^ + df)] and (b) ANOVA: *r* = √(SSm/SSt), SSm = between-group effect and SSt = total amount of variance in the data. According to Cohen, *r* = 0.10 represents a small effect size, r = 0.30 represents a medium effect size and r = 0.50 represents a large effect size [25].

Statistical analyses were performed using the statistics program SPSS version 20.0 (SPSS Inc.).

### Ethics, consent and permissions

The present study was approved by the IRB of the University Hospital of the Federal University of Juiz de Fora, Brazil. Participation was voluntary, informed consent was obtained (signed by students) after explaining the objectives and before the intervention, collected data were anonymized to maintain the integrity of the responders, and the data were handled and stored in accordance with the tenets of the Declaration of Helsinki (2008).

## Results

Of the 239 students in the three groups initially selected, 230 were assessed (6 absent and 3 refusals). This gave final samples of 72 for the “2014.1 Group” (Control Group - CG), 82 for the “2014.3 Group” (“Experiencing Aging” Group - EA) and 76 for the “2015.1 Group” (“Myths of Aging” Group – MA).

Concerning the baseline demographics, the EA Group has more females than the MA group (63.4% versus 39.5%, *p* = 0.008) and was younger (18.71 ± 1.43 years versus 19.73 ± 2.72 years, *p* = 0.009). However, no differences on family income between groups (55.1% had a family income of ≥ 7 minimum wages in the EA group and 50.0% had a family income of ≥ 7 minimum wages in the MA group).

We carried out the analysis of skewness and kurtosis for all instruments used and, based on the ranges of Gravetter (±2.00) [26], there was no evidence of skewness and kurtosis, ranging from −0.09 (SE: 0.16) to 1.08 (SE: 0.17) for skewness and −0.69 (SE: 0.32) to 1.50 (SE: 0.34) for kurtosis.

The students were assessed at baseline (prior to interventions, one week into the medical course) on knowledge about aging and attitudes toward older adults (Table 1). Subsequently, EA and MA groups were compared, where the CG served as post-intervention control for these intervention groups. At baseline, statistically significant differences were found only for basic cognitive knowledge in geriatrics (greater in EA Group, *p* = 0.004).

**Table 1:** Knowledge, attitudes and empathy at baseline among groups submitted to Experiencing Aging (ES) and Myths of Aging (MA) workshops

	Group	*N*	Mean	Standard Deviation	*r*	Standard Error	*p*
UCLA Total Pre (Attitudes)	EA	80	51.84	4.72	0.10	0.52	0.240
	MA	73	50.88	5.34		0.62	
Knowledge Total Pre	EA	82	4.70	1.49	0.23	0.16	**0.004**
	MA	76	4.00	1.46		0.16	
Palmore Positivism – positive opinions about aging	EA	78	58.07	6.16	0.07	0.69	0.383
	MA	71	57.18	6.30		0.74	
Palmore Total (correct responses) – Facts about aging	EA	82	11.61	2.74	0.09	0.30	0.248
	MA	76	11.13	2.41		0.27	
Maxwell-Sullivan – Total Attitude	EA	54	15.50	2.95	0.10	0.40	0.256
	MA	76	16.18	3.62		0.41	
Maxwell-Sullivan – Total Empathy	EA	54	5.31	1.62	0.02	0.22	0.804
	MA	76	5.41	2.37		0.27	

EA: Experiencing aging

MA: Myths of aging

The post-intervention analysis for the EA Group (Table 2) revealed the following changes: a significant difference on the UCLA scale total (a worse general attitude – *p* = 0.001), greater negativism on the Palmore questionnaire (negative opinions about aging) post intervention (*p* < 0.001), a worse attitude toward elderly people on the Maxwell-Sullivan attitude scale (*p* = 0.007) and an improvement in empathy toward the elderly on the Maxwell-Sullivan empathy scale (*p* = 0.001). No significant differences in students’ knowledge pre and post-intervention were observed.

**Table 2:** Difference pre- and post-intervention for EA and MA groups on Knowledge about aging, empathy, attitude and Palmore scale (facts on aging)^*^

	Mean	*N*	Standard Deviation	Standard Error	*r*	*s*
**Experiencing aging**						
UCLA Total Pre	51.88	78	4.67	0.53	0.36	**0.001**
UCLA Total Post	50.51	78	5.19	0.58		
Knowledge Total Pre	4.70	82	1.49	0.16	0.07	0.511
Knowledge Total Post	4.77	82	1.55	0.17		
Palmore Positive Pre	58.11	76	6.17	0.70	0.66	<**0.001**
Palmore Positive Post	52.72	76	6.40	0.73		
Palmore Total Pre	11.61	82	2.74	0.30	0.23	0.033
Palmore Total Post	10.94	82	2.28	0.25		
Attitude Total Pre	15.53	51	2.93	0.41	0.37	**0.007**
Attitude Total Post	16.78	51	2.83	0.39		
Empathy Total Pre	5.33	51	1.62	0.22	0.46	**0.001**
Empathy Total Post	4.63	51	1.31	0.18		
**Myths of aging**						
UCLA Total Pre	51.01	72	5.25	0.61	0.56	**<0.001**
UCLA Total Post	53.67	72	4.16	0.49		
Knowledge Total Pre	4.00	76	1.46	0.16	0.32	**0.004**
Knowledge Total Post	4.38	76	1.53	0.17		
Palmore Positive Pre	56.91	68	6.24	0.75	0.93	**<0.001**
Palmore Positive Post	72.14	68	3.83	0.46		
Palmore Total Pre	11.13	76	2.41	0.27	0.92	**<0.001**
Palmore Total Post	17.58	76	1.96	0.22		
Attitude Total Pre	16.17	75	3.65	0.42	0.47	**<0.001**
Attitude Total Post	14.83	75	3.46	0.40		
Empathy Total Pre	5.44	75	2.37	0.27	0.21	0.070
Empathy Total Post	5.13	75	2.10	0.24		

^*^We considered a *p* ≤ 0.0083 as significant (Bonferroni multiple comparison procedure)

The post-intervention analysis for the MA group (Table 2) revealed the following changes: a significant difference on the UCLA scale total pre- and post-intervention (an improvement in general attitude – *p* < 0.001), significant differences between students’ knowledge pre- and post-intervention (*p* = 0.003), marked improvement in score on the Palmore questionnaire, indicating a higher rate of correct responses on facts about aging (*p* < 0.001) and greater positivism (positive opinions about aging) post intervention (*p* < 0.001), and improvement in attitude toward elderly people on the Maxwell-Sullivan attitude scale (*p* < 0.0001). No improvement in empathy toward the elderly was found on the Maxwell-Sullivan empathy scale (*p* = 0.070).

Comparison of the groups post intervention (Table 3) revealed the following differences in total score: a significant difference on the UCLA scale total post-intervention between the MA x EA Groups (*p* = 0.001) and MA x CG (*p* < 0.001), and a marked improvement in knowledge score (Palmore questionnaire) for the MA group compared to the others (*p* < 0.001). The MA group exhibited a more positive view about elderly people than the other groups (*p* < 0.001) and also a better attitude toward older people on the Maxwell-Sullivan attitude scale. No significant differences were detected between the groups for students’ knowledge or for empathy on the Maxwell-Sullivan empathy scale. All significant results were maintained after carrying out ANCOVA with age and gender as covariates

**Table 3:** Comparison among groups post-intervention (total score on scales and post - pre difference)

	EA	CG	MA	*r*		
	M (SD)	M (SD)	M (SD)		*p*	Post-hoc^a^
UCLA Total Post	50.51 (5.19)	50.00 (5.68)	53.67 (4.18)	0.30	**<0.001**	EA x MA (*p* = 0.001); CG x MA (*p* < 0.001); EA x CG (*p* = 0.809)
Knowledge Total Post	4.77 (1.55)	4.64 (1.35)	4.38 (1.53)	0.11	0.256	EA x MA (*p* = 0.233); CG x MA (*p* = 0.544); EA x CG (*p* = 0.852)
Palmore Positivism	52.72 (6.40)	55.38 (5.29)	72.14 (3.83)	0.85	**<0.001**	EA x MA (*p* < 0.001); CG x MA (*p* < 0.001); EA x CG (*p* = 0.009)
Palmore Total Post	10.94 (2.28)	10.22 (2.24)	17.58 (1.96)	0.84	**<0.001**	EA x MA (*p* < 0.001); CG x MA (*p* < 0.001); EA x CG (*p* = 0.104)
Attitude Total Post	16.78 (2.83)	16.29 (4.2)	14.83 (3.46)	0.22	**0.007**	EA x MA (*p* = 0.009); CG x MA (*p* = 0.042); EA x CG (*p* = 0.734)
Empathy Total Post	4.63 (1.29)	5.32 (2.09)	5.13 (2.10)	0.14	0.140	EA x MA (*p* = 0.324); CG x MA (*p* = 0.824); EA x CG (*p* = 0.124)

^a^Results were maintained after carrying out ANCOVA with age and gender as covariates

Lastly, comparison of pre-post differences between the EA and MA groups (Table 4) revealed: a significant difference on the UCLA scale total, an improvement in score and positive view about elderly people on the Palmore questionnaire, and an improvement in attitude toward elderly people on the Maxwell-Sullivan attitude scale in the MA group compared to the EA Group. No significant difference between knowledge and empathy was noted.

**Table 4:** Comparison among groups post-intervention (post - pre difference)

	Group	*N*	Mean	Standard Error	*r*	*p*
UCLA Total (Post - Pre)	EA	78	−1.371	0.399	0.48	**<0.001**
	MA	72	2.652	0.466		
Knowledge Total (Post - Pre)	EA	82	0.073	0.110	0.14	0.071
	MA	76	0.381	0.129		
Palmore Positive (Post - Pre)	EA	82	−0.670	0.308	0.86	**<0.001**
	MA	76	6.447	0.317		
Palmore Correct (Post - Pre)	EA	76	−5.394	0.716	0.79	**<0.001**
	MA	68	15.235	0.752		
Attitude (Post - Pre)	EA	51	1.254	0.444	0.42	**<0.001**
	MA	75	−1.346	0.294		
Empathy (Post - Pre)	EA	51	−0.705	0.194	0.14	0.124
	MA	75	−0.306	0.166		

*We considered a *p* ≤ 0.0083 as significant (Bonferroni multiple comparison procedure)

## Discussion

The present study found that different brief educational strategies had a mixed effect (positive and negative) on the attitudes, knowledge and empathy toward elderly of first year medical students. Comparing the two strategies, the “Experiencing Aging” strategy was associated with increased empathy among the students, albeit with worsening attitude. The “Myths of Aging” strategy was associated with an improvement in overall attitude and a more positive view of aging and with no changes in empathy toward older adults.

These findings can be explained by the fact that in “Experiencing Aging”, students experience the functional limitation associated with aging [27–29], and thus are able to put themselves in the shoes of the elderly, thereby increasing their empathy. However, owing to the simulation of the limitations and disabilities (visual difficulty, disabilities, among others), it is likely that this worsened students’ attitudes toward the elderly, deeming them incapable of managing their lives due to the numerous limitations demonstrated in the activity. These results are similar to the findings of a previous investigation [30], but conflict with other studies that found positive or neutral results after using this same strategy [8, 31]. In our view, because this activity was introduced at the beginning of the course (students were not yet familiar with the subject and held many stereotypes regarding the elderly), it may have strengthened the myths of aging leading to the decline in attitude. Perhaps if implemented among students with greater knowledge in the area of geriatrics and gerontology, results may have been different, as suggested by some studies in doctors and students with more years of “experiencing aging” [32].

On the other hand, the “Myths of Aging” activity involves questions and answers whose main strategy is to demystify the stereotypes surrounding aging [33]. In this context, the students modify the way they imagine and see aging, which can result in a change in views on elderly. With regard to empathy, because the students had not experienced the limitations imposed by aging, no significant differences were evident, despite a tendency toward improvement. With regard to knowledge, an increase in the number of right answers to the facts on aging was noted, given that some content addressed in the “Myths of Aging” workshop can help answer the Palmore. Cognitive knowledge however, showed no changes, a situation which might be explained by the fact that this type of knowledge was not the focus of the workshop (the content of these questions were not taught in this activity).

These results contribute further evidence to this field of teaching. Despite the numerous studies addressing educational strategies for teaching geriatrics, results remain mixed and conflicting, where some systematic reviews have shown promising results [8, 31] whereas others have not [14]. One important finding revealed by the present study was the fact that teaching, can lead to mixed results, highlighting that strategies must be assessed and evidence-based.

Although a subject not extensively discussed in health education, other interventions in the gerontology area have also reported worsening attitudes, including one study using the same intervention employed in the present study. Henry et al. [30] assessed 156 health students using the Experiencing Aging activity (aging game), MacKnight [34] assessed 83 first year medical students after a home visit and van Zuilen et al. [35] assessed 288 junior and senior medical students after 2 weeks of a geriatrics course (which includes a rotation). All these studies found a worsening attitude towards older adults and suggest that exposure of students to only the unhealthy side of aging (experiencing limitations or exposure to chronic patients) can reinforce stereotypes of aging.

Based on this discussion, it is clear that strategies can have different effects on students, depending on when they are introduced, on the profile of students and the manner in which they are implemented. Thus, educators must establish the optimal strategy for meeting learning goals. In this context, some may argue that having a positive attitude towards older adults without seeing things from the perspective of the elderly (i.e. empathy), could not have a strong influence on future professional practice and then develop the attitude. We believe this reflection must be made and, in our opinion, perhaps a strategy combining the two workshops, demystifying the myths first and subsequently experiencing aging, may potentialize attitudes and empathy early in medical training.

The present study has several limitations. First, the study provided only a snapshot assessment of the students. It is unclear whether these results will persist over the long term. Second, this was an intervention-based, non-randomized study with a control group. Although constituting a limitation, this type of study is widely conducted in the area of medical education. Third, the investigation was conducted within a single Brazilian medical school, and so caution should be exercised when generalizing the results. Forth, there was a significant difference among groups concerning age and gender. The reasons for these differences are not clear, since the admittance criteria were the same through the years and the number of spots remained stable. Based on previous data, the demographics of our medical school classes can vary among semesters [36]. Nevertheless, in order to minimize these differences, we carried out the ANCOVA test including age and gender as covariates. Fifth, since we are carrying out multiple analyses (multiple comparisons), we decided to use the Bonferroni correction in order to reduce the type I error (a more conservative approach). This procedure could increased probability of making type II error, and consequently reduce statistical power.

Nevertheless, the study has also some strengths, such as an appropriate number of students, the use of interventions that have been previously tested by other research groups and the use of a relatively large number of internationally recognized scales, which allows the comparison with other countries.

Future studies should focus on how these strategies work in different cultures around the world, since some cultures have more stereotypes towards older persons. For instance, the present study found we found a mean of 3.7 in our pre-intervention sample, which is similar to US students with 3.7 [37] and to Singaporean students with 3.6 [38]. However, in regard to the Palmore test, US students have a higher score than Chinese students and our Brazilian students (16.1 versus 12.2 versus 11.6) [37, 39].

Other future directions of research are: (a) the investigation of strategies focusing on both empathy and attitude-enhancing activities, allowing students to become empathetic with their older patients, as well as, avoiding ageism and stereotypes towards aging, (b) in which moment these games may be delivered in order to students fully benefit from these strategies. A comparison between different moments of the course (beginning or final students) is warranted and (d) how these strategies could impact the long-term clinical practice and attitudes of these students.

### Conclusions

In conclusion, educational strategies can influence the attitudes and empathy of students differently, leading to both desirable and undesirable outcomes. These results underscore the importance of assessing educational strategies in medical teaching to ascertain in what manner, situations and settings these activities should be run.

## Additional file

Additional file 1: Study Questionnaire. (DOCX 87 kb)

## Abbreviations

ANCOVA: Analysis of covariance
ANOVA: Analysis of Variance
CG: Control group
EA: Experience aging
IRB: Institutional review board
MA: Myths of aging
r: Effect size
UCLA: University of California – Los Angeles
UFJF: Federal University of Juiz de For a
