# Supplementary material for: Experiencing aging or demystifying myths? – impact of different “geriatrics and gerontology” teaching strategies in first year medical students
Source: BMC Med Educ. 2017 Feb 8;17:35. doi: 10.1186/s12909-017-0872-9 (PMC5299654; doi:10.1186/s12909-017-0872-9)
Supplement: Additional file 1: — Study Questionnaire. (DOCX 87 kb) [file 12909_2017_872_MOESM1_ESM.docx]

**Study Questionnaire – English language version**

Gender

a) Male

b) Female

Age

______ years old

Family Income

R$ ____________ (R$ - “Reais” is the Brazilian currency)

Semester

( ) 1^st^  ( ) 7^th^

( ) 2^nd^ ( ) 8^th^

( ) 3^rd^ ( ) 9^th^

( ) 4^th^ ( ) 10^th^

( ) 5^th^ ( ) 11^th^

( ) 6^th^ ( ) 12^th^

**Facts about aging (Palmore-FAQ-1) - Multiple-choice version**

1. The proportion of people over 65 who are senile (have impaired memory, disorientation, or dementia) is:
2. about 1 in 100
3. about 1 in 10
4. about 1 in 2
5. the majority
6. The senses that tend to weaken in old age are:
7. sight and hearing
8. taste and smell
9. sight, hearing and touch
10. all five senses
11. The majority of old couples:
12. have little or no interest in sex
13. are not able to have sexual relations
14. continue to enjoy sexual relations
15. think sex is only for the young
16. Lung vital capacity in old age
17. tends to decline
18. stays about the same among non-smokers
19. tends to increase among healthy old people
20. is unrelated to age
21. Happiness among old people is:

a) rare

b) less common than among younger people

c) about as common as among younger people

d) more common than among younger people

1. Physical strength:
2. tends to decline with age
3. tends to remain the same among healthy old people
4. tends to increase among healthy old people
5. is unrelated to age
6. The percentage of people over 65 in long-stay institutions (such as nursing homes, mental hospitals, and homes for the aged) is about:
7. 5%
8. 10%
9. 25%
10. 50%
11. The accident rate per driver over age 65 is:
12. higher than for those under 65
13. about the same as for those under 65
14. lower than for those under 65
15. unknown
16. Most Workers over 65:
17. work less effectively than younger workers
18. work as effectively as younger workers
19. work more effectively than younger workers
20. are preferred by most employers
21. The proportion of people over 65 who are able to do their normal activities is about:
22. one-tenth
23. one-quarter
24. one-half
25. three-fourths
26. Adaptability to change among people over 65 is:
27. rare
28. present among about half
29. present among most
30. more common than younger people
31. As for old people learning new things:
32. most are unable to learn at any speed
33. most are able to learn, but at a lower speed
34. most are able to learn as fast as younger people
35. learning speed is unrelated to age
36. Depression is more frequent among:
37. people over 65
38. adults under 65
39. young people
40. children
41. Old people tend to react:
42. slower than younger people
43. at about the same speed as younger people
44. faster than younger people
45. slower or faster than younger people, depending on the type of test
46. Old people tend to be:
47. more alike than younger people
48. the same as younger people in terms of alikeness
49. less alike than younger people
50. more alike in some respects and less alike in others
51. Most old people say:
52. they are seldom bored
53. they are sometimes bored
54. they are often bored
55. life is monotonous
56. The proportion of old people who are socially isolated is:
57. almost all
58. about half
59. less than a fourth
60. almost none
61. The accident rate among workers over 65 tends to be:
62. higher than among younger workers
63. about the same as among younger workers
64. lower than among younger workers
65. unknown because there are so few workers over 65
66. The proportion of the Brazilian population now age 65 years or over is:
67. 5%
68. 10%
69. 20%
70. 30%
71. Medical practitioners tend to give older patients:
72. lower priority than younger patients
73. the same priority as younger patients
74. higher priority than younger patients
75. higher priority if they were from the SUS (Unified Brazilian Health System)
76. The poverty rate among old people is:
77. higher than among children under age 18
78. higher than among all persons under 65
79. about the same as among persons under 65
80. lower than among persons under 65
81. Most old people are:
82. employed
83. employed or would like to be employed
84. employed, do housework or volunteer work, or would like to do some kind of work
85. not interested in any work
86. Religiosity tends to be:
87. increase in old age
88. decrease in old age
89. be greater in the older generation than in the younger generations
90. be unrelated to age
91. Most old people:
92. are seldom angry
93. are often angry
94. are often grouchy
95. often lose their tempers
96. The health and economic status of old people (compared to younger people) in the year of 2030 will:
97. be higher than now
98. be about the same as now
99. be lower than now
100. show no consistent trend

**Modified Maxwell-Sullivan attitudes toward the elderly scale**

Attitude toward caring for the elderly

1. I will welcome elderly patients to my practice

a) strongly agree

b) agree

c) neither agree nor disagree

d) disagree

e) strongly disagree

2. Medical care of elderly can be done quickly

a) strongly agree

b) agree

c) neither agree nor disagree

d) disagree

e) strongly disagree

3. Wearisome to take care of elderly

a) strongly agree

b) agree

c) neither agree nor disagree

d) disagree

e) strongly disagree

4. Elderly need too much attention and sympathy

a) strongly agree

b) agree

c) neither agree nor disagree

d) disagree

e) strongly disagree

5. Treatment of elderly is hopeless

a) strongly agree

b) agree

c) neither agree nor disagree

d) disagree

e) strongly disagree

6. Elderly are not able to take care of their own needs

a) strongly agree

b) agree

c) neither agree nor disagree

d) disagree

e) strongly disagree

7. Elderly take medicines as prescribed

a) strongly agree

b) agree

c) neither agree nor disagree

d) disagree

e) strongly disagree

8. Treatment of elderly is too time consuming

a) strongly agree

b) agree

c) neither agree nor disagree

d) disagree

e) strongly disagree

Empathy to geriatric patients

1. I can truly empathize with older patients

a) strongly agree

b) agree

c) neither agree nor disagree

d) disagree

e) strongly disagree

2. I understand what it feels like to have problems with aging

a) strongly agree

b) agree

c) neither agree nor disagree

d) disagree

e) strongly disagree

3. Understanding my elderly patients is valuable to me

a) strongly agree

b) agree

c) neither agree nor disagree

d) disagree

e) strongly disagree

**UCLA Geriatric Attitudes Test**

1. Most old people are pleasant to be with

a) strongly disagree

b) somewhat disagree

c) somewhat neutral

d) agree

e) strongly agree

2. The federal government should reallocate money from Medicare to research on AIDS or pediatric diseases.

a) strongly disagree

b) somewhat disagree

c) somewhat neutral

d) agree

e) strongly agree

3. If I have the choice, I would rather see younger patients than elderly ones.

a) strongly disagree

b) somewhat disagree

c) somewhat neutral

d) agree

e) strongly agree

4. It is society’s responsibility to provide care for its elderly persons.

a) strongly disagree

b) somewhat disagree

c) somewhat neutral

d) agree

e) strongly agree

5. Medical care for old people uses up too much human and material resources.

a) strongly disagree

b) somewhat disagree

c) somewhat neutral

d) agree

e) strongly agree

6. As people grow older, they become less organized and more confused.

a) strongly disagree

b) somewhat disagree

c) somewhat neutral

d) agree

e) strongly agree

7. Elderly patients tend to be more appreciative of the medical care I provide than are younger patients.

a) strongly disagree

b) somewhat disagree

c) somewhat neutral

d) agree

e) strongly agree

8. Taking a medical history from elderly patients is frequently an ordeal.

a) strongly disagree

b) somewhat disagree

c) somewhat neutral

d) agree

e) strongly agree

9. I tend to pay more attention and have more sympathy towards my elderly patients than my younger patients

a) strongly disagree

b) somewhat disagree

c) somewhat neutral

d) agree

e) strongly agree

10. Old people in general do not contribute much to society

a) strongly disagree

b) somewhat disagree

c) somewhat neutral

d) agree

e) strongly agree

11. Treatment of chronically ill old patients is hopeless.

a) strongly disagree

b) somewhat disagree

c) somewhat neutral

d) agree

e) strongly agree

12. Old persons don’t contribute their fair share towards paying for their health care.

a) strongly disagree

b) somewhat disagree

c) somewhat neutral

d) agree

e) strongly agree

13. In general, old people act too slow for modern society.

a) strongly disagree

b) somewhat disagree

c) somewhat neutral

d) agree

e) strongly agree

14. It is interesting listening to old people's accounts of their past experiences.

a) strongly disagree

b) somewhat disagree

c) somewhat neutral

d) agree

e) strongly agree

**Basic knowledge in geriatrics**

1) Older patient presents a progressive cognitive impairment, visual hallucinations, bradykinesia and cognitive fluctuation. The first hypothesis for this patient should be:

(A) Progressive supranuclear palsy

(B) Frontotemporal dementia

(C) Dementia with Lewy bodies

(D) Parkinson's disease dementia

2) A 75-year-old patient, with no psychiatric history, was hospitalized due to a urinary tract infection. During the hospitalization, the patient became agitated, presented visual hallucination and was disoriented in time and space. The most probable diagnosis is:

(A) Alzheimer’s disease

(B) Delirium

(C) Vascular dementia

(D) Depression with psychosis

(E) Panic disorder

3) The development of a gradual onset and continuing memory impairment, at least ONE cognitive disturbance (aphasia, apraxia, agnosia and executive functioning), social and occupational function impairment, no significant anatomical changes in neuroimaging, no infectious or metabolic disorders and the disturbance is not better accounted for by another disorder (e.g., major depressive disorder, schizophrenia).

These are hallmark characteristics of:

(A) depression

(B) Progressive nonfluent aphasia

(C) Mild cognitive impairment

(D) Alzheimer’s disease

(E) Frontotemporal dementia

4) The following are considered potentially inappropriate medications in the elderly, with the exception of:

(A) tricyclic antidepressants (e.g. amitriptyline)

(B) antihistamines (e.g. Dexchlorpheniramine)

(C) antiarrhythmic drugs (e.g. amiodarone)

(D) benzodiazepines (e.g. diazepam)

(E) Angiotensin-converting enzyme (ACE) inhibitors (e.g. enalapril)

5) The prescription of medications in the elderly must be careful because:

(A) the increase in the fat mass and the reduction in total body water are responsible for increasing the half-life of liposoluble drugs and for decreasing the distribution of hydrosoluble drugs

(B) the high liver blood flow that happens with aging increases the metabolism of flow-dependent drugs

(C) The high activity of the P450 cytochrome that happens with aging increases the oxidative metabolism

(D) the increase of the kidney mass and renal flow with aging reduces the clearance for renally excreted drugs

6) The pharmacological treatment for Alzheimer’s disease is:

(A) Hypnotics

(B) Phytotherapeutic Drugs

(C) Anticholinergics

(D) Anti-cholinesterase drugs

(E) Antipsychotics

7) Concerning the treatment of the vascular dementia, please choose the correct answer:

(A) Gingko biloba has a proved positive effect in the cognitive impairment

(B) Nimodipine is indicated after an ischemic event.

(C) Hydergine® (ergoloid mesylates) is recommended for the long-term treatment

(D) The use of oral anticoagulants can prevent the vascular dementia

(E) Controlling risk factors such as hypertension and diabetes is the best preventive approach

8) In the initial stages of Alzheimer’s disease, we can early observe:

(A) Gait disorder

(B) Apraxia.

(C) Aphasia.

(D) Memory impairment

(E) Sleepiness

9) The comprehensive geriatric assessment (CGA):

(A) is better designed to use in non frail older persons

(B) uses multiprofessional teams and several assessment tools

(C) does not increase the number of diagnosis

(D) does not impact the institutionalization

10) Which of the following are used in the Katz Index of Independence in Activities of Daily Living:

(A) feeding, continence and taking medications correctly

(B) bathing, dressing and taking medications correctly

(C) be oriented in time and space, changing in position and toileting

(D) financing care, cooking and feeding.

(E) feeding, dressing and toileting
